# Correlation between lymphatic endothelial markers and lymph node status or N-staging of colorectal cancer

**DOI:** 10.1186/s12957-017-1276-3

**Published:** 2017-11-21

**Authors:** Xing-mao Zhang, Wen-xiao Han, Hong-ying Wang, Qiang He

**Affiliations:** 1 Department of Hepatobiliary Surgery, Beijing Chaoyang Hospital, Capital Medical University, Beijing, China; 2 State Key Laboratory of Molecular Oncology, Cancer Institute and Cancer Hospital, Chinese Academy of Medical Sciences, Peking Union Medical College, Beijing, China

**Keywords:** LYVE–1, VEGFR–3, Podoplanin, Prox–1, Colorectal cancer, RTQ–PCR

## Abstract

**Background:**

The purpose of this study is to examine the expression levels of lymphatic endothelial markers in colorectal cancer and to explore the correlation between the expression levels of markers and lymph node status.

**Methods:**

Forty-seven paired fresh tumor tissues and para-cancerous tissues were collected from colorectal cancer patients who received surgical treatment between August 2015 and March 2016 in Cancer Hospital, Chinese Academy of Medical Sciences. Real-time quantitative PCR (RTQ–PCR) was used to check the expression levels of LYVE–1, VEGFR–3, Podoplanin, and Prox–1 in tumor and para-cancerous tissues.

**Results:**

The positive expression rates of LYVE–1, VEGFR–3, Podoplanin, and Prox–1 in tumor tissues were 100, 93.6, 100, and 91.4%, but 100, 100, 100, and 87.2% in para-cancerous tissues. Comparing with para-cancerous tissues, tumor tissues had significantly lower expression levels of LYVE–1 (*P* < 0.001) and VEGFR–3 (*P* = 0.013) and higher levels of Podoplanin (*P* = 0.016) and Prox–1 (*P* = 0.078). There was no correlation between lymph node status and the expression level of LYVE–1 in tumor tissues (*P* = 0.354) or par-cancerous tissues (*P* = 0.617); similar results were found for VEGFR–3 (*P* = 0.631, 0.738), Podoplanin (*P* = 0.490, 0.625), and Prox–1 (*P* = 0.503, 0.174). Meanwhile, there was no correlation between N-staging and the expression level of LYVE–1 in tumor tissues (*P* = 0.914) or para-cancerous tissues (*P* = 0.784); similar results were found for VEGFR–3 (*P* = 0.493, 0.955), Podoplanin (*P* = 0.199, 0.370), and Prox–1 (*P* = 0.780, 0.234).

**Conclusions:**

There was no correlation between expression levels of lymphatic endothelial markers and lymph node status; LYVE–1, VEGFR–3, Podoplanin, and Prox–1 could not be used for predicting the lymph node status or N-staging of colorectal cancer.

## Background

Lymphatic system markers which have been used for studying the mechanism of lymphatic metastasis for several kinds of malignant tumors recently include lymphatic endothelial markers and lymphatic endothelial growth factors, and the lymphatic endothelial markers mainly include lymphatic vessel endothelial hyaluronic acid receptor–1 (LYVE–1), vascular endothelial growth factor receptor 3 (VEGFR–3), Podoplanin, and Prox–1 [1, 2]. The correlation between the lymphatic endothelial markers and lymphatic metastasis in some malignant tumors such as gastric cancer, breast cancer, ovarian cancer, and so on have been studied by some centers, and there is no consensus yet whether lymphatic endothelial markers can be used for predicting the N-staging of carcinoma [3–7]. In this study, we used real-time quantitative PCR (RTQ–PCR) to examine the expression of the four kinds of lymphatic endothelial markers in colorectal cancer; meanwhile, we analyzed the difference of expression levels in tumor tissue and para-cancerous tissue and we also analyzed the correlation between the expression levels of LYVE–1, VEGFR–3, Podoplanin, or Prox–1 and lymph node status.

## Methods

### Tissue collection

Forty-seven paired fresh tumor tissues and para-cancerous tissues (2-cm tissue adjacent to cancer) were collected from colorectal cancer patients who received surgical treatment between August 2015 and March 2016 in Cancer Hospital, Chinese Academy of Medical Sciences. All tissues were collected within half an hour after the removal of specimens. RNA was extracted using the standard RNAzol procedure. cDNA was subsequently synthesized using a reverse transcription kit according to the manufacturer’s instructions.

### Primers

Primers of four markers were synthesized by Sangon Biotech (Shanghai) Co., Ltd: LYVE–1 forward primer: 5′–AAGAATGAAGCTGCTGGGTTT–3′, LYVE–1 reverse primer: 5′–GACATAGCAAAATCCAAGACCA–3′; VEGFR–3 forward primer: 5′–AGGGAGACGCCCTTTCATG–3′, VEGFR–3 reverse primer: 5′–GAGGGCTCTTTGGTCAAGCA–3′; Podoplanin forward primer: 5′–CACGGAGAAAGTGGATGGAGA–3′, Podoplanin reverse primer: 5′–GCCGATGGCTAGTAAGACCC–3′; Prox–1 forward primer: 5′–AAAGCAAAGCTCATGTTTTTTTATA–3′, Prox–1 reverse primer: 5′–GTAAAACTCACGGAAATTGCTAAA–3′.

### RTQ–PCR

Bio–Rad CFX96 real-time PCR system was used for RTQ–PCR; relative quantitative analysis was proceeded according to the SYBR Green I method. Reaction system was as follows: SsoFastTM EvaGreen supermix 10 μl, forward primer of target gene 2 μl (2 μM), reverse primer 2 μl (2 μM), deionized water 4 μl, and cDNA 2 μl. RTQ–PCR conditions were as follows: 40 cycles, pre-degeneration 95 °C for 30′, degeneration 95 °C for 10′, and annealing extension 60 °C for 10′.

### Statistical analysis

Statistical analyses were performed using statistical software package SPSS version 16.0. A *P* value less than 0.05 was considered to be statistically significant. Student’s *t* test was used for the analysis of continuous variables, and one-way ANOVA was used for the analysis between groups.

## Results

Twenty-eight of the 47 patients were males and 19 were females in this study. Their ages ranged from 34 to 84 with the median age of 56.3. The number of patients with ascending colon cancer, transverse colon cancer, descending colon cancer, sigmoid colon cancer, and rectal cancer were 7, 2, 1, 8, and 29, respectively. Lymph node involvement was detected in 27 patients. The general parameters of patients were shown in Table 1.

**Table 1:** The general parameters of patients in this study

Parameters	
Gender, case	
Male	29
Female	18
Age, year, mean (range)	56.3 (34–84)
Location of tumors, case	
Cecum	0
Ascending colon	7
Transverse colon	2
Descending colon	1
Sigmoid colon	8
Rectum	29
Tumor size, cm, mean (range)	4.8 (2.2–14.0)
Type of pathology, case	
Adenocarcinoma	44
Mucinous adenocarcinoma	1
Signet ring cell carcinoma	2
Degree of differentiation, case	
Well	3
Moderate	34
Poor	10
Depth of invasion, case	
T2	2
T3	38
T4a	7
N-staging, case	
N0	20
N1a	7
N1b	5
N2a	8
N2b	7
TNM staging, case	
IIa	18
IIb	2
IIIb	18
IIIc	9

### Different expressions of the four markers between tumor tissues and para-cancerous tissues

#### Expression rates in tumor tissues and para-cancerous tissues

Both of the positive expression rates of LYVE–1 in tumor tissues and para-cancerous tissues were 100%, and the same results could be found for Podoplanin. A 93.6% positive expression rate of VEGFR–3 was found in tumor tissue, but 100% in para-cancerous tissue. Prox–1 had a positive expression rate of 91.4% in tumor tissue and 87.2% in para-cancerous tissue.

#### Expression levels in tumor tissues and para-cancerous tissues

**Fig. 1:**
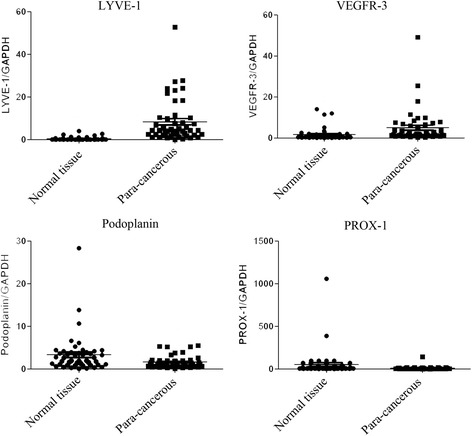
Expression levels of markers in tumor tissues and para-cancerous tissues. Both LYVE-1 and VEGFR-3 had a significantly higher expression level in para-cancerous tissues than that in tumor tissues (LYVE-1: *P* < 0.001; VEGFR-3: *P* = 0.013), while Podoplanin had a lower expression level in para-cancerous tissues than in tumor tissues (*P* = 0.016); no significantly different expression levels between tumor tissues and para-cancerous tissues was found for Prox–1

Expression level of LYVE–1 in para-cancerous tissues was significantly higher than that in tumor tissues (*P* < 0.001), and a higher expression level VEGFR–3 was also found in para-cancerous tissues compared with that in tumor tissues (*P* = 0.013), whereas a different result was found for Podoplanin, the higher expression level was checked in tumor tissues (*P* = 0.016). For Prox–1, there were no significantly different expression levels in between tumor tissues and para-cancerous tissues (Fig. 1).

### The correlation between lymph node status and expression levels of markers

**Fig. 2:**
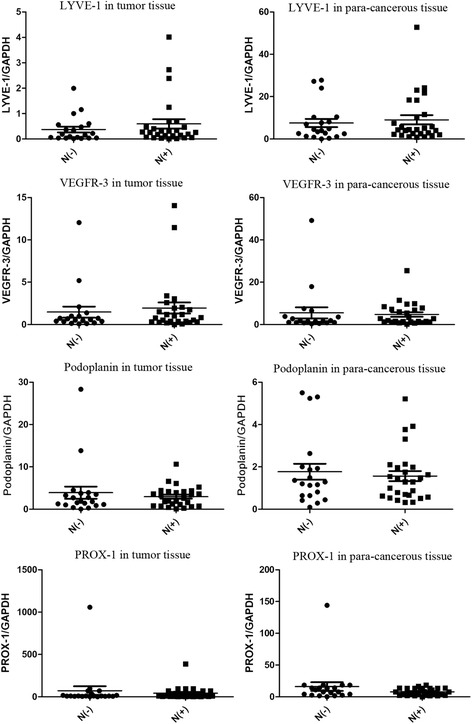
Correlation between lymph node status and expression levels of markers in tumor tissues or para-cancerous tissues. Status of lymph node did not influence the expression level of the four makers; no significantly different expression levels were found in tumor tissues and no obvious differences in para-cancerous tissues. (In tumor tissues: LYVE-1, *P* = 0.354; VEGFR–3, *P* = 0.631; Podoplanin, *P* = 0.490; Prox–1, *P* = 0.503; in para-cancerous: LYVE-1, *P* = 0.617; VEGFR–3, *P* = 0.738; Podoplanin, *P* = 0.625; Prox–1, *P* = 0.174)

The expression level of LYVE–1 in tumor tissues of patients with lymph node involvement was similar with that in patients without lymph node involvement (*P* = 0.354), and the similar results were found for VEGFR–3 (*P* = 0.631), Podoplanin (*P* = 0.490), and Prox–1 (*P* = 0.503). Similarly, the expression level of LYVE–1 in para-cancerous tissues of N(+) staging patients was similar with that in N(−) staging patients (*P* = 0.617); the similar results were found for VEGFR–3 (*P* = 0.738), Podoplanin (*P* = 0.625), and Prox–1 (*P* = 0.174) (Fig. 2).

### The correlation between N-staging and expression level of markers

**Fig. 3:**
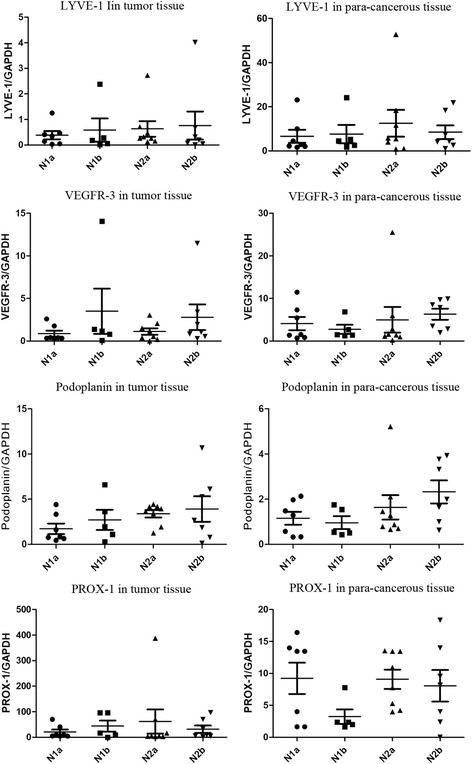
Correlation between N-staging and expression levels of markers in tumor tissues or para-cancerous tissues. The expression levels of these four makers in tumor tissues or para-cancerous tissues were not impacted by N-staging of tumor. (In tumors tissues: LYVE-1, *P* = 0.914; VEGFR–3, *P* = 0.493; Podoplanin, *P* = 0.199; Prox–1, *P* = 0.780; in para–cancerous: LYVE-1, *P* = 0.784; VEGFR–3, *P* = 0.955; Podoplanin, *P* = 0.370; Prox–1, *P* = 0.234)

The difference of expression level of LYVE–1 in tumor tissues of patients with N1a, N1b, N2a, and N2b staging was not found (*P* = 0.914); same results were found for VEGFR–3 (*P* = 0.493), Podoplanin (*P* = 0.199), and Prox–1 (*P* = 0.780). No difference was found for LYVE–1 in para-cancerous tissues (*P* = 0.784); similarly, no differences for VEGFR–3 (*P* = 0.955), Podoplanin (*P* = 0.370), and Prox–1 (*P* = 0.234) (Fig. 3).

## Discussion

The discovery of lymphatic system markers is promoting the research progress of metastatic mechanism. The correlation between lymphatic endothelial markers and lymphatic metastasis had been reported by several centers during the last decade. However, there is no consensus yet whether lymphatic endothelial markers can be used for predicting lymphatic status.

LYVE–1, as a new hyaluronic acid receptor, was found in 1999 [8]. It mainly expressed in lymphatic endothelial cells [9, 10]. Some studies showed that lymphatic endothelial cells of malignant tumors had specific expression of LYVE–1 [11]. Expression of LYVE–1 in gastric cancer was studied by Ozmen et al. [12]; his results showed that the expression level of LYVE–1 in cancer tissues was obviously higher than that in para-cancerous tissues, and an increased expression level was related with the increased proportion of lymph node involvement. In 2006, Gao et al. [13] designed a study to examine the expression of LYVE–1 in colorectal cancer tissues and normal tissues; his results showed that the expression level of LYVE–1 in cancer tissues was higher than that in normal tissues although no significant difference was found. A PCR study designed by Lu et al. [14] confirmed that LYVE–1 mainly existed in margins of tumor.

Among all lymphatic endothelial markers, VEGFR–3 was discovered earliest. VEGFR–3 has vital function during the process of generation and development of vascular system [15, 16]. A study designed by Kawakami et al. [5] found that the positive rate of VEGFR–3 in colorectal cancer tissues was 79.2%; meanwhile, his study confirmed that there was no correlation between the expression level of VEGFR–3 in cancer tissues and lymph node involvement; however, different conclusion was made by some other professors: some studies showed that an increased rate of lymph nodes involvement was related to the higher expression level of VEGFR–3 in colorectal cancer tissues [17, 18].

Initially, the Podoplanin was found on the surface of glomerular podocytes, and its expression in lymphatic endothelial cells was firstly reported in 1999 [19]. Podoplanin mainly exists in small lymphatic vessels, but it does not express in big lymphatic vessels with smooth muscle. Braun et al. [20] found that Podoplanin was a sensitive factor for predicting the involvement of lymph nodes in invasive breast cancer; the study of Wada et al. [21] demonstrated that the expression level of Podoplanin was related to the involvement rate of lymph nodes and lymphovascular invasion in T1 stage colorectal cancer.

Prox–1, as one kind of nuclear transcription factors, is the homologous gene of prospero got from *Drosophila melanogaster*; it has the functions of regulating cell mitosis and inducing the differentiation of lymphatic endothelial cells [22]. Parr et al. [23] found that colorectal cancer tissues had a higher expression level of Prox–1; the study results of Agarwal et al. [7] showed that breast cancer with lymph node involvement had a higher expression level of Prox–1 in cancer tissues.

Some similar or different results were found based on our study; all of the four markers had the high positive expression rates not only in tumor tissues but also in para-cancerous cancer tissues, and the expression levels of LYVE–1 and VEGFR–3 was higher in para-cancerous tissues than that in tumor tissues, whereas the higher expression levels of Podoplanin and Prox–1 was found in tumor tissues. There was no correlation between lymph node status and the expression level of LYVE–1 in tumor tissues or para-cancerous tissues; similar results were found for VEGFR–3, Podoplanin, and Prox–1. Meanwhile, no correlation between N-staging and the expression level of LYVE–1 in tumor tissues or para-cancerous tissues; similar results were found for VEGFR–3, Podoplanin, and Prox–1.

## Conclusions

There was no correlation between lymph node status and expression of LYVE–1, VEGFR–3, Podoplanin, or Prox–1; these four lymphatic endothelial markers could not be used for predicting the lymph node status or N-staging of colorectal cancer.

## Abbreviations

LYVE–1: Lymphatic vessel endothelial hyaluronic acid receptor–1
VEGFR–3: Vascular endothelial growth factor receptor 3
